# High-Field fMRI Reveals Brain Activation Patterns Underlying Saccade Execution in the Human Superior Colliculus

**DOI:** 10.1371/journal.pone.0008691

**Published:** 2010-01-13

**Authors:** Ruth M. Krebs, Marty G. Woldorff, Claus Tempelmann, Nils Bodammer, Toemme Noesselt, Carsten N. Boehler, Henning Scheich, Jens-Max Hopf, Emrah Duzel, Hans-Jochen Heinze, Mircea A. Schoenfeld

**Affiliations:** 1 Department of Neurology, Otto-von-Guericke-University, Magdeburg, Germany; 2 Leibniz-Institute for Neurobiology, Magdeburg, Germany; 3 Center for Cognitive Neuroscience, Duke University, Durham, North Carolina, United States of America; 4 Department of Psychiatry, Duke University, Durham, North Carolina, United States of America; 5 Kliniken Schmieder, Allensbach, Germany; Rutgers University, United States of America

## Abstract

**Background:**

The superior colliculus (SC) has been shown to play a crucial role in the initiation and coordination of eye- and head-movements. The knowledge about the function of this structure is mainly based on single-unit recordings in animals with relatively few neuroimaging studies investigating eye-movement related brain activity in humans.

**Methodology/Principal Findings:**

The present study employed high-field (7 Tesla) functional magnetic resonance imaging (fMRI) to investigate SC responses during endogenously cued saccades in humans. In response to centrally presented instructional cues, subjects either performed saccades away from (centrifugal) or towards (centripetal) the center of straight gaze or maintained fixation at the center position. Compared to central fixation, the execution of saccades elicited hemodynamic activity within a network of cortical and subcortical areas that included the SC, lateral geniculate nucleus (LGN), occipital cortex, striatum, and the pulvinar.

**Conclusions/Significance:**

Activity in the SC was enhanced contralateral to the direction of the saccade (i.e., greater activity in the right as compared to left SC during leftward saccades and vice versa) during both centrifugal and centripetal saccades, thereby demonstrating that the contralateral predominance for saccade execution that has been shown to exist in animals is also present in the human SC. In addition, centrifugal saccades elicited greater activity in the SC than did centripetal saccades, while also being accompanied by an enhanced deactivation within the prefrontal default-mode network. This pattern of brain activity might reflect the reduced processing effort required to move the eyes toward as compared to away from the center of straight gaze, a position that might serve as a spatial baseline in which the retinotopic and craniotopic reference frames are aligned.

## Introduction

The ability to perform saccadic eye movements to shift the observer's gaze to an object or location of interest is a fundamental and critical function of humans and other animals. The neural underpinnings of such eye movements involve the activation of a network of cortical and subcortical structures that leads to a precise discharge of activity in the muscles around the eye to align the observer's fovea with the object of interest [1]. One of the key regions for the initiation and coordination of eye movements is the superior colliculus (SC), a layered subcortical structure that forms the tectum of the midbrain [for a review see 1], [2]. The SC appears to function as a critical hub for the control of eye movements, which is facilitated by it being involved in the integration of diverse sensory and attention-related signals, including inputs from the retina, visual cortex, frontal eye fields (FEF), supplementary eye fields (SEF), parietal cortex, and thalamic structures [1], [3]–[8].

Several decades of animal research on the SC have provided a great body of knowledge about the contribution of this subcortical brain structure to the control of eye movements and to the orienting of attention [2], [9]–[18]. The superficial layers of the SC have been found to process visual information that arrives from the retina, visual cortex, and FEF [e.g.], [3], [19,20]. Visual-processing neurons in these superficial layers are topographically organized, with the visual field input being predominantly represented in the contralateral SC [21]. The deep layers of the SC, on the other hand, receive divergent sensory, motor, as well as higher cortical area input, and neuronal activity in these layers has been associated with the initiation of eye- and head-movements [9], [10] and with shifts of attention [12], [17] to selected target stimuli. Electrical microstimulation of deep-layer neurons elicits saccadic eye movements to the contralateral visual field, with a specific direction and amplitude corresponding to a well-defined spatial topographic map [5], [10], [14], [18], [22].

In humans, although there have been various neuroimaging studies on the cortical areas involved in saccade execution [23]–[33], the investigation of the SC during the execution of saccadic eye movements has been limited to a few reports [25], [34]–[36], mostly due to methodological challenges like insufficient spatial resolution and low signal-to-noise ratio for this small and deeply located subcortical brain region. However, there are several studies that investigated the sensitivity of the SC to visual stimulation in the absence of eye movements [37]–[42]. In line with animal research, these studies showed that the SC in humans is more responsive to visual stimuli in the contralateral versus ipsilateral visual field and, moreover, that its activity can be modulated by attention [39], [41]. However, the contralateral predominance in the human SC for the execution of endogenously cued saccades has not yet been reported.

The main goal of the current study was to carry out a high-resolution (7-Tesla) examination of the activity pattern evoked during saccadic eye movements in the human SC, with a particular focus on whether the underlying activity pattern exhibits a contralateral predominance. Subjects performed saccades to the left or the right as cued by a color change at central fixation. From a methodological perspective, it is beneficial to disentangle activity related to traditional saccades away from central fixation (centrifugal saccades) and activity during saccades that are necessary to return the gaze to central fixation between trials [centripetal saccades or “return]-[saccades”, e.g., 32,43]. We thus used a paradigm with separate cues for centrifugal and centripetal saccades, thereby enabling us to separately examine the brain activity related to the two saccade types. In order to optimize the signal estimation within the SC we estimated an alternative model, in addition to the standard hemodynamic response function (HRF) model, using an HRF with an earlier peak that has been demonstrated to be better-suited for investigating SC activity [42]. We hypothesized that both saccade types (centrifugal and centripetal) would be associated with a contralateral predominance as reflected by enhanced fMRI activity levels within the SC contralateral to saccade direction. Furthermore, we were interested in potential differences regarding the general activity level during centrifugal and centripetal saccades.

## Materials and Methods

### Subjects and paradigm

Ten healthy right-handed subjects participated in the study (mean age ± standard deviation SD: 27±2.5, 5 female). One subject had to be excluded due to high levels of artefact in the anatomical scan. All participants were recruited from the student population of the Otto-von-Guericke University in Magdeburg. The experimental protocols were approved by the ethics committee of the University of Magdeburg, Faculty of Medicine, and all participants gave written informed consent to participate in accordance with the Declaration of Helsinki. All subjects underwent a clinical neurological examination before the fMRI scan.

**Figure 1:**
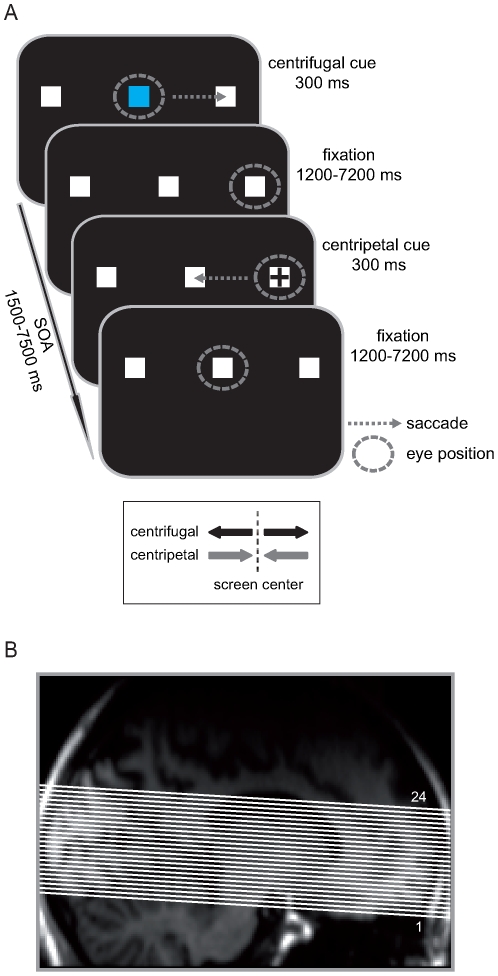
Paradigm and fMRI-acquisition volume. (**A**) Subjects performed saccades away from (centrifugal) and towards (centripetal) the screen center. Centrifugal saccades were cued by a central color change (300 ms), symbolically indicating the direction of the saccade (e.g., blue equals right vs. green equals left). After each centrifugal saccade, the gaze remained at the designated lateral square until a black cross indicated to perform a centripetal saccade back to the center. In fixation trials, indicated by a third cue color (e.g., red), subjects' gaze remained at the center position. The stimulus onset asynchrony (SOA) was jittered between 1500 and 7500 ms for all centrifugal and centripetal cues to facilitate the deconvolution of the event-related fMRI responses. (**B**) Functional images were acquired as a partial-head volume covering the upper brainstem including the SC, as well as large portions of the occipital and prefrontal cortex. The layout of the 24 functional image slices is superimposed on a single-subject's T1-weighted anatomical scan.

We employed an event-related design systematically manipulating both the direction of the saccade (i.e., left vs. right) and its relation to head-centered space (i.e., centrifugal vs. centripetal). The visual display consisted of a screen with three white squares (each of 0.5° degree of visual angle) at a distance of 8° on black background with the central square located at the center of the screen (see Fig. 1A). At the beginning of each trial, a colored cue (300 ms) appeared at the center position, indicating either the direction to which the saccade should be performed (e.g., blue means to perform a saccade to the right vs. green means to the left) or that the subject should maintain fixation at the center (e.g., red). After the execution of a centrifugal saccade to either the left or right white square, the subjects' gaze remained at the new location until a black cross (300 ms) was presented there, cuing the return-saccade to the center square (centripetal saccade). Subjects were asked to execute each saccade as quickly and as accurately as possible and to try not to blink throughout saccade execution.

The onsets of all cues (centrifugal and centripetal) were pseudo-randomly varied in timing with a stimulus onset asynchrony (SOA) of 1500 to 7500 ms to allow for effective event-related BOLD response estimation [44]. The instructional meaning of the different cue colors was counterbalanced across subjects.

### fMRI data acquisition

Prior to actual scanning, subjects performed a short training session to get familiarized with the task. Inside the scanner subjects performed 6 experimental runs, each of five-minute duration, resulting in a total of 110 trials in each condition (i.e., centrifugal left, centrifugal right, centripetal left, centripetal right, maintain fixation). fMRI images were acquired using a 7 Tesla Magnetom MRI scanner (Siemens, Erlangen, Germany) with a standard head-coil system. Each functional run consisted of 250 volumes with 24 T2*weighted echo planar slices (EPIs; TR = 1500 ms, TE = 24 ms, FoV = 224 mm, matrix size of 160*160 yielding a voxel size of 1.4*1.4*2 mm) acquired as a partial-head volume in an axial slice orientation using an interleaved scanning order (see Fig. 1B). To achieve the high spatial resolution with single shot EPI acquisition, parallel imaging (GRAPPA) with an acceleration factor of two and a partial Fourier acquisition scheme (75%) were applied. The functional data was corrected online for motion artifacts during each run using a scanner-implemented correction sequence. In addition, susceptibility-induced distortions were corrected by applying a method based on local point-spread functions [45].

T1-weighted anatomical whole-head images (MP-RAGE sequence, matrix size 320*320 yielding a voxel size of 0.7*0.7*2 mm) were acquired to enable coregistration and normalization. In addition, a T2-weighted turbo spin echo sequence with hyper-echoes was used to acquire anatomical images with the same slice position and slice orientation as the functional partial-head volumes (matrix size 256*256 yielding a voxel size of 0.9*0.9*2 mm), facilitating the localization of the SC in relation to other midbrain areas. In order to control for the correct execution of the cued saccades, eye movements were monitored online throughout all runs using a pupil tracking system [46].

### Data analysis

Images were preprocessed and analyzed using Statistical Parametric Mapping (SPM5; Wellcome Department of Imaging Neuroscience, University College, London, UK). In order to equalize extreme intensity gradients caused by the high field strength, an image mask was derived based on the subjects' individual T1-weighted anatomical scans by adjusting the intensity threshold. This mask retained the structural information of the T1-weighted image and could be utilized for defining normalization parameters. The anatomical images were normalized to a voxel size of 1*1*1 mm. Functional EPIs were corrected for acquisition delay and co-registered to the original T1-weighted image. After spatial normalization to a final voxel size of 2*2*2 mm, functional images were smoothed with an isotropic 4-mm full-width half-maximum Gaussian kernel. Before model estimation, a high-pass temporal filter of 128 seconds was applied [47].

A standard two-stage mixed-effects model [48] was used for statistical analysis. In the first stage, blood-oxygen level-dependent (BOLD) responses were modeled by delta functions at the stimulus onsets for the five event types of interest (i.e., centrifugal left, centrifugal right, centripetal left, centripetal right, maintain fixation), which were then convolved with a standard hemodynamic response function (HRF) to form covariates of a general linear model [GLM, 48]. A recent study has demonstrated that the BOLD signal within the SC is best represented by an HRF peaking between 4 and 5 seconds [42]. In order to optimize the signal estimation within the main area of interest in the present study, i.e., the SC, an additional GLM was estimated using an alternative HRF that peaked at 4.5 seconds (referred to as *4.5sec-model*) as compared to the standard HRF peaking at 6 seconds (referred to as *6sec-model*). For both models, contrast images of the individual subjects were entered into a random effects analysis using one-sample T-tests for voxel-wise comparisons (significance threshold p = .005 and voxel-extent threshold k = 15). Coordinates of significant voxel clusters are reported in a standard stereotactic reference space (MNI, Montreal Neurological Institute) and functional overlays are displayed on the average of the subjects' spatially normalized T1-weighted images (a detailed description of activation clusters under both models is provided Tables 1 and 2).

**Table 1:** Regions exhibiting activation during both centrifugal and centripetal saccades versus fixation trials.

region	L/R	local maxima peak coordinates (MNI)			T-value
		*x*	*y*	*z*	
***6sec-model:***					
***all saccades>fixation***					
	L	−2	−80	2	13.46
medial occipital cortex	R	12	−72	0	10.22
LGN	R	22	−30	−2	9.37
anterior occipital cortex	L	−16	−66	0	8.22
lateral occipital cortex	L	−34	−78	0	5.60
anterior occipital cortex	R	12	−60	4	5.45
putamen	R	26	2	10	5.28
pulvinar	L	−18	−32	8	5.03
pulvinar	R	24	−30	6	4.75
putamen	L	−24	−4	6	4.41
***4.5sec-model:***					
***all saccades>fixation***					
medial thalamus	R	8	−22	4	5.57
lateral occipital cortex	L	−42	−68	2	5.20
SC	L	−2	−28	−6	5.01
anterior occipital cortex	L	−16	−68	0	5.57
SC	R	2	−26	−6	4.87
anterior occipital cortex	R	14	−62	2	4.83
medial occipital cortex	R	12	−72	9	4.82
medial occipital cortex	L	−6	−90	−2	4.81
medial thalamus	L	−4	−18	0	4.73
LGN	R	24	−28	0	4.46
LGN	L	−18	−32	0	4.33
putamen	L	−18	14	6	4.26

L: left hemisphere; R: right hemisphere.

MNI: Montreal Neurological Institute.

T-value: local maxima thresholded at p = .005, extent threshold k = 15.

**Table 2:** Regions exhibiting differential activations in the direct comparison between centrifugal and centripetal saccades.

region	L/R	local maxima peak coordinates (MNI)			T-value
		*x*	*y*	*z*	
***6sec-model:***					
***centrifugal>centripetal***					
anterior occipital cortex	L	−22	−52	−2	6.47
fusiform gyrus	R	20	−42	−12	4.73
LGN	L	−24	−34	2	4.48
SC	R	6	−26	−6	4.12
***centripetal>centrifugal***					
insula	L	−38	−14	2	6.46
parietal cortex	L	−60	−46	0	5.65
lateral PFC	L	−40	42	−4	5.45
insula	R	42	−10	4	5.08
mPFC	L	−4	48	−2	4.87
lateral PFC	R	38	36	−4	4.43
hippocampus	L	−28	−14	−20	4.11
mPFC	R	8	48	10	3.27
***4.5sec-model:***					
***centrifugal>centripetal***					
SC	L	−8	−28	−2	8.26
SC	R	8	−24	−2	5.77
medial thalamus	R	6	−16	14	5.57
anterior insula	L	−30	18	6	5.56
inferior frontal gyrus	R	30	−24	−4	5.26
anterior insula	R	36	18	4	4.88
medial thalamus	L	−2	−22	6	3.39
***centripetal>centrifugal***					
*(no significant activations)*					

L: left hemisphere; R: right hemisphere.

MNI: Montreal Neurological Institute.

T-value: local maxima thresholded at p = .005, extent threshold k = 15.

To verify the voxel-wise statistics in an orthogonal fashion, anatomically defined regions of interest (ROIs) were established for the SC representing the main area of interest for the present study. In order to achieve a precise anatomical outline for the SC that was entirely independent from any functional activation, spherical ROIs (average radius of 3 mm) were derived from the subject's T1-weighted images with regard to the individual neuronanatomy and intensity differences using the MRIcron tool (http://www.sph.sc.edu/comd/rorden/mricro.html). A similar ROI analysis was performed regarding the medial prefrontal cortex (mPFC), since this region showed a robust deactivation in the voxel-wise analysis that was object to further validation. In the mPFC case, spherical ROIs with a radius of 3 mm were centered based on the local deactivation maxima of the functional activity across all subjects derived from the orthogonal contrast *‘all saccades versus fixation’* (left mPFC: x y z = −4 44 −2, right mPFC: x y z = 8 46 14). For both the SC and mPFC ROIs, the parameter estimates of the response amplitudes (beta values) based on the respective SPM model were extracted for the event-related response for each condition (*centrifugal right, centrifugal left, centripetal right, centripetal left, and fixation*) using the MarsBar region of interest analysis toolbox [49], [50]. Note that the ROI analysis within the SC was based on the *4.5sec-model* that used an HRF peaking at 4.5 seconds, whereas the ROI analysis within mPFC was based on the *6sec-model* using the standard HRF peaking at 6 seconds. The extracted parameter estimates reflecting the response amplitude were analyzed via a 3-way repeated measures ANOVA (rANOVA), with the factors *saccade direction* (left vs. right), *side* (left vs. right hemisphere), and *saccade type* (centrifugal vs. centripetal). In addition, the estimates for each saccade condition (centrifugal and centripetal; collapsed across leftward and rightward saccades) were compared to those during fixation via paired T-tests. To inspect the actual shape of the BOLD response in both ROIs, we extracted the time course for each ROI and condition based on a shape-assumption-free finite-impulse-response (FIR) model.

## Results

### Brain activations during saccade execution

**Figure 2:**
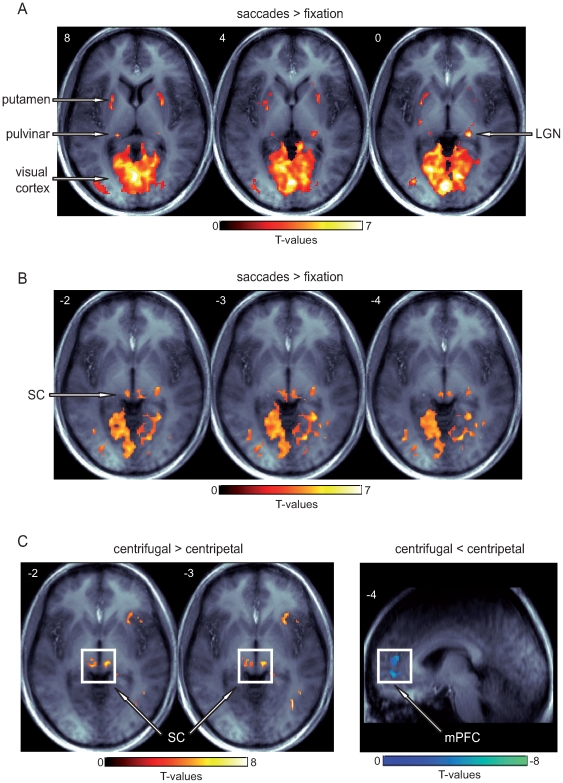
Saccade-related BOLD activity. (**A**) Saccade execution was associated with robust activity in bilateral medial occipital cortex, putamen, pulvinar, and LGN as compared to cued fixation trials based on the standard HRF model (*6sec-model*). (**B**) The same contrast derived from the alternative *4.5sec-model* (i.e., optimized for the SC) revealed robust saccade-related activity within both the left and right SC. (**C**) The direct comparison between centrifugal and centripetal saccades revealed higher activity within the SC (*4.5sec-model*) as well as a stronger prefrontal deactivation for the former (*6sec-model*). Activations are displayed on the averaged T1-weighted image. Display cut off: T>2.5, extent threshold k>15.

Representative slices of the acquired partial-head volume are shown in Figure 2 displaying the activated brain regions during saccade execution (see Tables 1 and 2 for an activation cluster overview under both models; the T-values refer to the local maxima of significant activation clusters thresholded at p = .005, extent threshold k = 15). The analyses of the voxel-wise statistical maps based on the standard *6sec-model* comparing all saccades to fixation revealed a common network of saccade-related regions, including bilateral occipital visual cortex close the calcarine sulcus (T-values: left T = 13.5, right T = 10.2) as well as right LGN (T = 9.4), bilateral putamen (left T = 4.4, right T = 5.3), and bilateral pulvinar (left T = 5.00, right T = 4.8; see Fig. 2A and Table 1). The identical contrast based on the alternative *4.5sec-model* revealed robust bilateral SC activity (left T = 5.0, right T = 4.9; see Fig. 2B and Table 1). Note that several saccade-related regions were also significantly activated using the alternative *4.5sec-model*, however, most local maxima were lower as compared to the standard *6sec-model*. The only region that displayed higher activity in the *4.5sec-model* as compared to the *6sec-model* in addition to the SC was the medial thalamus (left T = 4.7, right T = 5.6).

A direct comparison between centrifugal and centripetal saccades (see Fig. 2C and Table 2) revealed an enhanced BOLD response in the SC during centrifugal as compared to centripetal saccades (local activity maxima based on the *4.5sec-model*: left SC T = 8.3, right SC T = 5.8). The same contrast revealed a stronger deactivation within bilateral medial prefrontal cortex (mPFC, see Fig. 2C, right panel) during the execution of centrifugal saccades as compared to centripetal saccades (local activity maxima based on the *6sec-model*: left mPFC T = −4.9, right mPFC T = −3.3). The location of this deactivation close to the midline corresponds to the prefrontal part of the default-mode network, which is known to exhibit activity deactivations during demanding attentional tasks.

### ROI Analyses of Parameter Estimates

**Figure 3:**
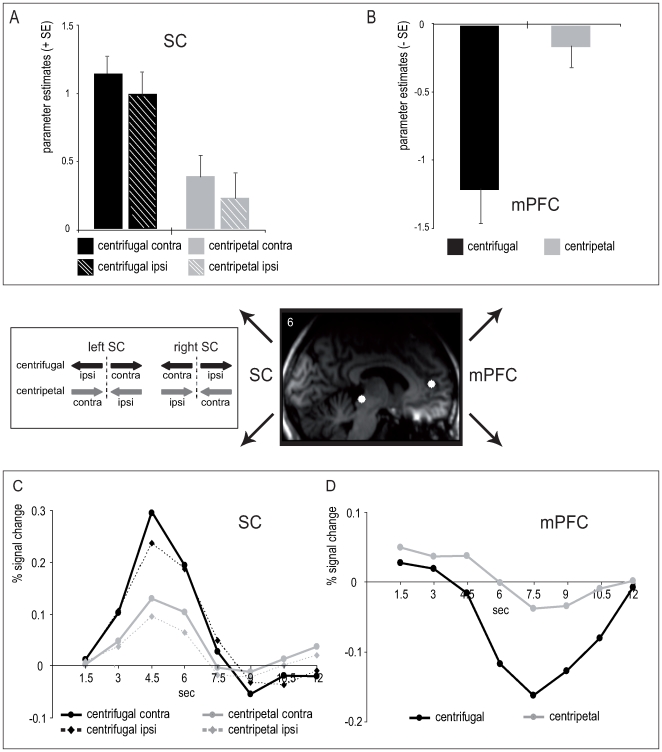
ROI-based parameter estimates and FIR time courses. (**A**) The analysis of parameter estimates (beta values) revealed enhanced activity in the left SC during saccades into the right visual field and vice versa, as well as enhanced SC activity for centrifugal as compared to centripetal saccades. Data are collapsed across hemispheres displaying collicular activity contralateral (contra) and ipsilateral (ipsi) to the saccade direction. Leftward saccades are considered to be contralateral with respect to the right SC, and ipsilateral with respect to the left SC. Similarly, rightward saccades are considered contralateral and ipsilateral with respect to the left and right SC, respectively. (**B**) At the same time, centrifugal saccades were associated with a robust prefrontal deactivation that was significantly smaller during centripetal saccades. Data are collapsed across hemispheres and saccade direction. The corresponding ROI-based time courses closely match the differences in parameter estimates within SC (**C**) and mPFC (**D**). ROIs were defined orthogonally to condition-specific patterns, i.e. anatomically regarding the SC and on the basis of the contrast *‘all saccades versus fixation’* within mPFC. Representative ROI locations are displayed on a single-subject's T1-weighted image in the center. Error bars in panels A and B depict the standard error (SE) in each condition across subjects.

To provide orthogonal comparisons for all experimental conditions and to investigate the hypothesized contralateral properties of the SC, we extracted the parameter estimates reflecting the mean response amplitudes from the anatomically defined SC ROI based on the *4.5sec-model* (see Methods section for details). These activity estimates were analyzed by means of a 3-way repeated-measures ANOVA (rANOVA), with the factors *saccade direction* (left vs. right), *side* (left vs. right hemisphere), and *saccade type* (centrifugal vs. centripetal). The rANOVA of parameter estimates within the SC revealed a significant interaction between *saccade direction* and *side* (F_(1,8)_ = 8.94, p = .017), reflecting stronger activations ***contralateral*** to the direction of the saccade (Fig. 3A). Furthermore, we observed a main effect of *saccade type* (F_(1,8)_ = 11.51, p = .009), with higher activity for centrifugal compared to centripetal saccades (Fig. 3A), confirming the difference observed in the voxel-wise activation contrasts. The direct comparison of saccade trials (collapsed across leftward and rightward directions) relative to fixation trials revealed that SC activity was significantly smaller during fixation as compared to centrifugal saccades (T_(8)_ = 3.86, p = .005) while not significantly different from centripetal saccades (T_(8)_ = 1.84, p = .1).

In order to investigate the activity differences within the prefrontal default-mode network observed in the voxel-wise comparison, an analogous ROI-based analysis was performed within mPFC. As in the SC, the analysis revealed a main effect of *saccade type* (F_(1,8)_ = 15.82, p = .004), but in this case the effect was due to a stronger ***deactivation*** during centrifugal saccades as compared to centripetal saccades (Fig. 3B). During fixation, there was a smaller deactivation within the mPFC as compared to centrifugal saccades (T_(8)_ = 5.38, p = .001), but no significant difference between fixation and centripetal saccades (T_(8)_ = .23, p = .8). No other significant main effects or interactions were observed in the ROI-based analyses (p-values>.1).

To further inspect the shape characteristics of the BOLD response, the FIR time courses were extracted from the defined ROIs within SC and mPFC. In the SC, the resulting time courses resembled the predicted shape peaking at 4.5 seconds and confirmed the contralateral enhancement as well as the enhanced activity for centrifugal compared to centripetal saccades (Fig. 3C). With regard to the mPFC, the extracted FIR time course activity also confirmed the differential activity dependent on the saccade type, thus underscoring the inverted response shape (i.e., deactivation) of the event-related activity in this region that was specifically pronounced during centrifugal saccades (Fig. 3D). The matching results of the parameter estimates (beta values derived from both HRF models) and the FIR-based time courses underscore the robustness of the signal in the acquired data and confirm the choice of specific peak latencies for different regions (e.g., SC and mPFC).

## Discussion

### Contralateral predominance within the SC

In this study, we used 7-Tesla high-field fMRI to investigate brain areas involved in the execution of saccadic eye movements, with a specific focus on the role of the SC. Our findings demonstrate the predominantly contralateral functional representation of the generation of saccades in the human SC (i.e., the generation of leftward saccades is associated with greater activity in the right SC as compared to the left SC, and vice versa) that has been repeatedly shown in animal single-unit research [2], [14], [21]. Furthermore, the results extend previous investigations of visual stimulus processing in the human SC that employed visual stimulation paradigms [38], [39], [41], [42] and saccade tasks [25], [35], [36] by showing that the contralateral bias is also exhibited during endogenously cued saccades in the absence of exogenous attentional capture by peripheral stimuli. It seems likely that the observed bias towards the contralateral visual field in the present data is reflecting processes involved in both voluntary target selection and saccade execution. This pattern is consistent with findings of numerous animal studies showing an enhancement of neuronal activity within the contralateral SC prior to the execution of saccades [e.g., 21], as well as during covert attentional shifts in the absence of any eye movement [e.g., 17]. It should be noted that even with the high-field fMRI resolution available here, it was not possible to disentangle the different layers within the SC that are mainly involved in target selection from the layers that are more involved in saccade initiation. Most probably, however, the observed collicular BOLD response is based on neuronal activity from both sub-areas, those involved in saccade target selection and those areas with topographically organized visuomotor neurons that discharge time-locked to the onset of saccades into the contralateral visual field [e.g., 51].

With respect to a framework in which attention is both influenced by bottom-up salience and top-down relevance, the current paradigm clearly emphasizes the top-down component by using central instructional cues rather than peripheral salient stimuli to trigger the saccade [6], [8]. Here, subjects needed to interpret the meaning of the instructional cue and actively decide where to direct their attention and consequently move their eyes to. The planning of endogenously cued saccades thus involves a greater need for integration of information from higher cortical regions (i.e., for the color-direction mapping) as compared to exogenously triggered saccades [6].

Since the superficial layers of the SC are sensitive to characteristics of the visual stimulation itself, it is important to consider whether the contralateral enhancement might have been due to changes in the visual input prior to or after saccade execution, rather than from the saccade generation *per se*. Several considerations argue against this possibility. First, for the centrifugal saccades the pre-saccadic visual input did not differ for the left and right visual field, and thus there was no visual input difference that could have led to any contralateral predominance. Secondly, in regards to any contribution from the post-saccadic visual input, a slightly greater part of the visual input (i.e., the three placeholder squares; see Fig. 1) appeared in the visual field contralateral to the direction of the executed centrifugal saccade. However, this asymmetry would have enhanced activity ipsilateral to the saccade direction (i.e., saccades to the left would lead to slightly greater visual input in the right visual field, triggering activity in left SC as well as left occipital cortex). With regard to centripetal saccades, we can not exclude entirely that differences in the pre-saccadic visual input might have contributed to the contralateral bias, since in this case the pre-saccadic visual input would be expected to be slightly larger in the targeted visual field. Nevertheless, this would not explain the presence of a contralateral bias for both centrifugal and centripetal saccades. Furthermore, regarding the time of actual saccade execution, visual input is highly suppressed [52]. Taken together, it seems rather unlikely that slight differences in visual input could be the main source of the contralateral bias observed in the SC. It should be noted, however, that the onset of the central cue itself contributes to the observed SC signal, albeit in a non-lateralized fashion. This paradigmatic difference to most visual stimulation paradigms investigating the SC [e.g., 42] might result in a weaker contralateral predominance in the present study since the fMRI signal is representing both the sensory response to the cue (non-lateralized) as well as the mainly contralateral saccade initiation. Since the scanner room contains no light sources except for the stimulation screen itself, the illumination differences at the end of the stimulation screen and the beginning of the bore are minimal and unlikely to contribute significantly to the observed activity pattern. Moreover, all saccades are executed within a range of 16° of visual angle around the center of the screen leaving 10° of black screen to the edge of the bore. Regarding the robust occipital activity along the calcarine, similar activity levels have been reported for saccades in complete darkness [32], suggesting that visual input is not the main source of the observed occipital BOLD response. However, it remains possible that the visual display and the screen-edge luminance contribute to changes in occipital activity, specifically within the anterior parts of the calcarine representing eccentric parts of the visual field.

In that the SC has also been linked to the control of head movements, it is worth considering possible influences of small head movements to the present results. In this context, single-unit recordings in monkeys and cats have shown that a subpopulation of SC neurons discharges during head movements contralateral to the recording site and that the stimulation of those neurons triggers contraversive head movements [53], [54]. However, it seems unlikely that small head movements could have contributed significantly to the activity patterns reported here. Subjects' heads were fixed rather tightly within the scanner head coil, and subjects had been instructed and trained to not move their heads during scanning. Moreover, head movements were continuously monitored online during the experiment. Any movements of more than a few millimeters would have resulted in major artifacts in the fMRI images which were not observed. Even very small movements, which can produce imaging artifacts, are detectable by the imaging software, and these were corrected for. Most importantly, such very small head movements are unlikely to exhibit a systematic influence on collicular activity during different saccade conditions in the current experiment. One study directly compared eye movements alone, head movements alone, and gaze movements (combined eye- and head-movements) up to 14° of visual angle using fMRI [25]. The authors found no significant difference in SC activity between the three movement types, suggesting that the common mechanism that serves both eye- and head-movements does not result in a summation of effects regarding the collicular BOLD response.

In theory, saccade-related collicular activity could be influenced by differential activity overlap from trial to trial. Unlike covert attention shifts, the execution of an eye movement, besides requiring processing related to generating the eye movement itself, necessarily resets the fixation point and changes the visual field input [e.g., 55]. Moreover, subjects need to perform an active return-saccade to the center of straight gaze between trials that would likely invoke additional, and perhaps different, neural activations than the preceding centrifugal eye movement [43]. While studies using event-related potential (ERP) brain activity measures are able to identify and temporally separate initial-saccade activity and return-saccade activity based on the oculomotor signal, with fMRI the signal from such activity is certainly carried over onto the successive trial if not specifically dealt with. We therefore employed an experimental design, in which the event-related activity elicited by saccades away from fixation (centrifugal) would not be affected by activity overlap stemming from the saccade that re-centers the eyes (centripetal). All saccades (centrifugal and centripetal), as well as the fixation period control event were separately cued, with their onsets optimally jittered for an effective HRF estimation and deconvolution of activity overlap [44]. In addition to the exclusion of activity overlap between trials, the current paradigm permitted us to analyze the neural activity related to centrifugal and centripetal saccades separately. Importantly, the contralateral predominance within the SC was independent of the saccade type (i.e., centrifugal versus centripetal). This finding is consistent with the view that saccade vectors are coded with regard to the current visual field (contralateral representation) and thus independent of the relation between orbital position and body axis [1], [21].

### Differential activation patterns for centrifugal and centripetal saccades

In addition to the clear contralateral predominance for both saccade types, we observed robust differences in the general collicular activity level for centrifugal versus centripetal saccades, with greater activity for the former. What might be the possible sources of the observed differences between centrifugal and centripetal saccades? Given that the amplitude and direction properties of the motor-execution saccade vectors do not differ between these saccade types, it seems unlikely that the differences in collicular activity reflect differences in pure motor control output functions of the SC. Rather, it seems more likely that the observed higher activity for centrifugal saccades reflects increased processing demands during the preparation of the saccades. Such demands could be related to greater computational complexity for the initiation of centrifugal saccades and a corresponding increase in the allocation of attentional resources. The idea of increased task-related attentional demands is supported by the corresponding deactivation pattern observed in the medial prefrontal cortex. This mPFC area is not specifically related to the oculomotor system, but has been reported to be a core structure of the default-mode network, a set of areas that has been shown to exhibit deactivations with increased attentional demands [56]–[58]. Thus, the enhanced deactivation of the mPFC during the execution of centrifugal versus centripetal saccades would be quite consistent with higher processing demands.

One possible reason to consider for these observed collicular and prefrontal activity difference between the two saccade types is a difference in the directional predictability of the saccades. While the direction of centrifugal saccades away from central fixation in the current experiment (i.e., left versus right) was entirely unpredictable, for centripetal saccades the direction of the next movement was 100-percent predictable (i.e., back to central fixation). However, previous human neuroimaging studies have reported that the spatial as well as the temporal predictability of saccades is associated with enhanced rather than reduced levels of cortical saccade-related activity [i.e.], [FEF], [30,31,33]. In addition, a study using scalp-recorded ERPs reported higher frontal activity for centrifugal compared to centripetal saccades. Importantly, this was also the case when the timing and the saccade direction were both self-paced by the subject and did therefore not differ in their predictability [59]. With regard to the SC, we are not aware of any study showing differential activity depending on saccade predictability in humans. However, animal research has shown that the anticipation of a target location (spatial predictability) as well as the anticipated onset (temporal predictability) leads to increased baseline activity that helps to lift the transient firing of SC neurons above threshold [60]–[65]. Based on the above considerations, it seems unlikely that the differences in predictability in the present study can entirely account for the enhanced collicular activity during centrifugal saccades, although this possibility can not be ruled out on the basis of the current data.

Alternatively, the differential collicular activity pattern could be related to influences of the saccade-vector orientation relative to head-centered space. While centrifugal saccades always start from the center coordinate here, centripetal saccades necessarily start from an eccentric orbital position and return to the center of straight gaze. During straight gaze, eye-centered (retinotopic) and head-centered (craniotopic) reference frames are perfectly aligned in a head-restrained experimental setting. It has been previously shown behaviorally that saccades towards the center of straight gaze are facilitated, as reflected by shorter saccadic reaction times [66]. Studies investigating saccade-related SC activity in animals have demonstrated that the information about the pre-saccadic orbital position is integrated during collicular saccade programming and gave rise to the notion that this position signal might contribute to the so-called “re-centering bias” [e.g.], [ 67], [68]–[72]. More specifically, it has been suggested that saccades of identical direction and amplitude might require different levels of effort depending on the initial eye position [70], [72]. For example, if the orbital position deviates to the left from the gaze center, it has been postulated that less activity may be required in the left SC to perform a saccade to the right (i.e., in centripetal direction) than performing an analogous rightward saccade that starts at the gaze center (i.e., in centrifugal direction). From an evolutionary perspective it has been argued that the re-centering bias subserves the fast reorientation of the gaze towards the most convenient gaze coordinate in a changing visual environment–with respect to both muscular and attentional efficiency [73], [74]. A possible mechanism for the facilitation of re-centering (centripetal) saccades might be related to dynamic gain field modulations [e.g., 75] in collicular movement neurons during fixation, prior to the saccade, at positions away from the gaze center [70], [72]. Such a mechanism would predict an activity change in collicular neurons from low (center position) to higher (eccentric position) for centrifugal and from high to lower for centripetal saccades. However, this pattern would likely result in a difference in the baseline activity levels (i.e. prior to the saccade) as a function of fixation location, an effect that we did not observe in the ROI-based FIR model (Fig. 3C). It is possible that such an eye-position signal, which has been shown with single-unit recordings, just may not have ramified into a measurable fMRI signal. Regardless, however, the observed differential pattern appeared to be time-locked to the saccade relative to a baseline that did not differ for the two saccade types. This would suggest that the observed event-related activity mainly reflects the transient BOLD signal at the time of the saccade onset rather than any differential pre-saccadic activity due to fixation position.

### Comparing saccade-related activity to fixation trials

Animal studies have demonstrated that eye movements are processed by a dynamic interplay between movement neurons in the intermediate layer of the SC that exhibit burst activity time-locked to the saccade initiation and rostral neurons that stabilize the gaze during fixation [15], [64], [76]. Consequently, the actual saccade initiation should be mostly reflected by neuronal activity in intermediate collicular layers, while maintaining fixation should be mostly reflected by activity at the rostral pole. The distinction between these different collicular sub-regions using fMRI in humans is very challenging, however, and the observed BOLD response has to be regarded as a summed signal across different neuron types within this small brainstem structure.

Nevertheless, given that the execution of any saccade would be expected to be reflected in enhanced collicular activity, the observation that centrifugal but not centripetal saccades were associated with significantly higher activity as compared to fixation might seem paradoxical at first glance. In this context, however, it should be noted that the fixation condition we used in the current paradigm was not an entirely passive one. Since fixation trials were randomly intermixed with saccade trials, subjects were still required to interpret the color cue and respond adequately, i.e., either to execute a saccade in the indicated direction or to maintain fixation. Since the visual cues for saccade and fixation trials were physically equivalent, the onset of the cue is likely to result in similar activity of visual neurons in the superficial layers of the SC [2]. In contrast, activity associated with the actual saccade initiation is likely to be reflected in distinct collicular sub-regions as compared to maintaining fixation [1], [64]. In addition, fixation-neuron activity might have been especially enhanced in fixation trials to overcome the impulse of moving the eyes after the presentation of a cue, since most cues in the current paradigm request eye movements. Regardless, given the spatial resolution of fMRI, even at the high field strength employed here, we were not able to distinguish any topographical differences associated with saccade initiation versus fixation. Thus, when considering the observed BOLD signal as the summed activity across different collicular neurons, it seems likely that the relatively small saccade-related activity during centripetal saccades simply did not sufficiently exceed the average activity during fixation trials in order to be detectable with our fMRI recordings.

### Summary and conclusion

In summary, the current study provides several important findings regarding the neural underpinnings of saccadic eye movements in humans. Most importantly, it directly demonstrates for the first time the predominantly contralateral functional neuroanatomy of the human SC during saccade generation, observed in the absence of any sensory stimulation or attentional capture by peripheral salient stimuli. Accordingly, it contributes to a cross-species and cross-methodological validation of the functional neuroscience of this critical brainstem structure. In addition, the observed differential activity pattern for centrifugal versus centripetal saccades within the SC is consistent with the idea that the center of straight gaze, in which retinotopic and craniotopic reference frame are precisely aligned, might represent an efficient spatial reference position for eye movements from which the visual world can be explored.
